# Exosomal long noncoding RNA HOTTIP as potential novel diagnostic and prognostic biomarker test for gastric cancer

**DOI:** 10.1186/s12943-018-0817-x

**Published:** 2018-02-27

**Authors:** Rui Zhao, Yanli Zhang, Xin Zhang, Yongmei Yang, Xin Zheng, Xiaohui Li, Yingjie Liu, Yi Zhang

**Affiliations:** 1 Department of Clinical Laboratory, Qilu Hospital, Shandong University, 107 Wenhua Xi Road, Jinan, Shandong 250012 China; 2 Department of Clinical Laboratory, Shandong Provincial Third Hospital, Jinan, Shandong 250012 China

**Keywords:** Gastric cancer, Long noncoding RNA, HOTTIP, Diagnosis, Prognosis

## Abstract

Long noncoding RNA HOTTIP plays important roles in the generation and progression of human cancers. Exosomes participate in cellular communication by transmitting moleculars between cells and are regarded as suitable candidates for non-invasive diagnosis. However, the existence of HOTTIP in the circulating exosomes and the potential roles of exosomal HOTTIP in gastric cancer (GC) was poorly understood. This study aims at investigating the clinical roles of exosomal HOTTIP in GC. Serum exosomal HOTTIP from 246 subjects (126 GC patients and 120 healthy people) were detected by reverse transcription real-time quantitative polymerase chain reaction (RT-qPCR). Our results showed that expression levels of exosomal HOTTIP were typically upregulated in GC than in normal control (*P* < 0.001). And its expression levels were significantly correlated with invasion depth (*P* = 0.0298) and TNM stage (*P* < 0.001). The AUC for exosomal HOTTIP was 0.827, which demonstrated a higher diagnostic capability than CEA, CA 19–9 and CA72–4 (AUC = 0.653, 0.685 and 0.639, respectively) (*P* < 0.001). The Kaplan–Meier analysis showed a correlation between increased exosomal HOTTIP levels and poor overall survival (OS) (logrank *P* < 0.001). And univariate and multivariate COX analysis revealed exosomal HOTTIP overexpression was an independent prognostic factor in GC patients (*P* = 0.027). These findings demonstrated that exosomal HOTTIP may be a potential biomarker for GC in diagnosis and prognosis.

**Electronic supplementary material:**

The online version of this article (10.1186/s12943-018-0817-x) contains supplementary material, which is available to authorized users.

Gastric cancer (GC) is a huge burden worldwide with high morbidity and high mortality, especially in Eastern Asia [1]. Despite many advances in the diagnosis and treatment, the prognosis of GC patients remains poor, with over 70% patients ultimately died from this disease [2]. Early diagnosis and early treatment is conducive to improving prognosis of GC. The gold standard for GC, endoscopy followed by pathological examination, is invasive and uncomfortable [3]. Body fluids-based testing is considered to be a satisfactory method without invasion, but current serum tumor biomarkers used for GC diagnosis, like CEA, CA 19–9 and CA 72–4, have a low positive rate [4]. These realities highlight the necessity to discover other novel biomarkers with high specificity and high sensitivity.

Long non-coding RNAs (lncRNAs) are a group of non-coding RNAs with a length longer than 200 nucleotides [5]. Evidences show disorderly regulation of lncRNAs takes part in human disease [6]. The HOXA transcript at the distal tip (HOTTIP), which transcribed from the 5′ tip of HOXA cluster, has gained growing attention among cancer-related lncRNAs [7]. HOTTIP primarily targets WDR5/MLL complexes across HOXA via directly binding the adaptor protein WDR5, leading histone H3 lysine 4 trimethylation and gene transcription of several 5’ HOXA genes and reports have showed HOTTIP can promote cell proliferation through p21 inhibition or miRNA silencing [8–10]. Ye et al. [11] found that HOTTIP was significantly overexpressed in GC cell lines and downregulation of HOTTIP would inhibit cell proliferation, promote cell apoptosis, and degrade cell migration and invasion. Studies of GC tissues revealed that upregulated expressions of HOTTIP was associated to poor differentiation, positive lymph nodes metastasis and advanced clinical stage [12]. However, no study of HOTTIP in GC serum has been reported.

Exosomes are regarded as an entirely new mode of cell-to-cell communication with a size of 50-150 nm in diameter, which are secreted by many cells and can reflect the characteristics of their parent cells [13, 14]. Exosomes contain various and numerous cargos, including enzymes, proteins, lipids and RNAs, and present in perhaps all biological fluids, thus they are believed to participate in multiple pathological conditions, including cancer [15, 16]. Furthermore, exosomal double layer membrane is an ideal shelter for protecting the cargoes from being degraded and the stability and longevity of the RNA molecules are ideal for non-invasive diagnosis of tumor [17]. In previous study, our lab had proved the existence and stability of exosomal lncRNA in serum and detailed the clinical significance of exosomal lncRNA CRNDE-h for CRC [17]. In view of these observations, we supposed that GC cells are able to generate and release exosomes and the examination of exosomal HOTTIP may provide a noninvasive method for GC detection, patients subpopulation, prognosis prediction and even allow better understanding of etiopathology. In this study, we systematically investigated the expression of exosomal HOTTIP in GC serum and tapped information may be provided about the GC status. Then we further tested the feasibility that HOTTIP as a potential novel marker for GC diagnosis and prognosis and compared the diagnostic capability of HOTTIP with traditional biomarkers, including CEA, CA 19–9 and CA 72–4.

## Results and discussion

### Expression of exosomal HOTTIP and its relationship with clinicopathological festures in GC patients

In this study, we found that exosomal HOTTIP could be detected in culture medium of GC SGC7901 cell line, and the levels were increased with the incubation time extended (Additional file 1: Figure S1), suggesting exosomal HOTTIP was directly released from GC cells.

Then, we enrolled 126 GC patients and 120 normal healthy subjects between January, 2011 and May, 2012 at Qilu Hospital, Shandong University, China. Serum samples were collected before subjects had received any surgery, chemotherapy or radiation therapy. And then, they were sub-packed into Eppendorf tubes without Rnase, Dnase or pyrogen after being centrifuged at 11000 rpm for 10 min and immediately frozen and stored at − 80 °C until analysis. Levels of exosomal HOTTIP in these 246 serum samples were detected by RT-qPCR using GAPDH and UBC as internal control. We found that exosomal HOTTIP expression levels were evidently upregulated in GC patients (*P* < 0.001) (Additional file 2: Figure S2). To observe the stability of exosomal HOTTIP in serum, we measured their expression levels after treated with prolonged exposure to room temperature or mutiple freeze-thaw cycles and our results demonstrated that all these treatment had no influence on their expression (Additional file 3: Figure S3).

We also analyzed the association between exosomal HOTTIP expression levels and GC patients’ clinicopathological features. We found exosomal HOTTIP expression levels were positively associated with invasion depth (*P* = 0.0298) and TNM stage (*P* < 0.001) of GC. Conversely, there was no significant correlation of exosomal HOTTIP expression with other clinical features, such as age, gender, size of tumor, pathological differention and lymph nodes metastasis (all *P* > 0.05) (Table 1).

**Table 1:** Correlation between exosomal HOTTIP and clinicopathological parameters of gastric cancer (*n* = 126)

Features	Number of cases	Exosomal HOTTIP levels	*P* value
Age (years)			
< 60	61	2.348 (1.607–3.061)	0.1340
≥ 60	65	2.020 (1.423–2.891)	
Gender			
Female	60	2.194 (1.837–3.109)	0.0919
Male	66	2.166 (1.333–2.837)	
Tumor size (cm)			
< 5	63	1.995 (1.412–2.840)	0.1051
≥ 5	63	2.409 (1.826–3.039)	
Pathological differention			
Well + moderate	57	1.997 (1.573–3.164)	0.8273
Poor	69	2.419 (1.458–2.921)	
Invasion depth			
T1 + T2 + T3	78	2.109 (1.515–2.609)	0.0298*
T4	48	2.665 (1.535–4.269)	
Lymph nodes metastasis			
Negative	49	2.020 (1.508–2.973)	0.4204
Positive	77	2.348 (1.664–2.944)	
TNM stage			
I and II	55	1.624 (1.316–2.066)	< 0.001***
III and IV	71	2.782 (2.219–3.361)	

Data presented as median (25–75% interquartile range)

* *P* < 0.05, *** *P* < 0.001

### Diagnostic value of exosomal HOTTIP for GC patients

**Fig. 1:**
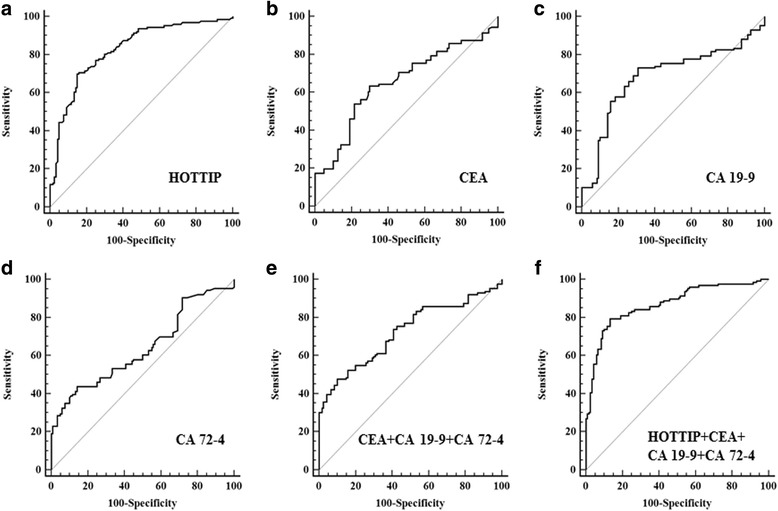
The ROC curves of biomarkers. **a** The ROC curve of exosomal HOTTIP. The area under the curve was 0.827. **b** The ROC curve of CEA. The area under the curve was 0.653. **c** The ROC curve of CA 19–9. The area under the curve was 0.685. **d** The ROC curve of CA 72–4. The area under the curve was 0.639. **e** The ROC curve of combine CEA, CA 19–9 and CA 72–4. The area under the curve was 0.723. **f** The ROC curve of combine CEA, CA 19–9, CA 72–4 and exosomal HOTTIP. The area under the curve was 0.870. *P* < 0.001

CEA, CA 19–9 and CA 72–4 are widely used in screening and auxiliary diagnosing gastrointestinal malignancies [4]. We measured their expression levels in GC serum by electrochemiluminescence method on Cobas E601 (Roche Diagnostics GmbH, Germany) and constructed ROC curves to evaluate and compared the diagnostic capacity of serum biomarkers. The area under the curve (AUC) for exosomal HOTTIP was 0.827 (Fig. 1a), which significantly higher than the AUCs for CEA, CA 19–9, CA 72–4 with 0.653, 0.685 and 0.639, respectively (*P* < 0.001; Fig. 1b, c and d). We calculated the combinative diagnostic value of CEA, CA 19–9 and CA 72–4, the AUC was 0.723 (Fig. 1e). And the results also showed the AUC for exosomal HOTTIP was significantly larger than that for these union (*P* < 0.001), indicating that exosomal HOTTIP was superior to them in GC diagnosis. We further evaluated the combinative diagnostic value of CEA, CA 19–9, CA 72–4 and exosomal HOTTIP for GC. The AUC for this combination was 0.870 (Fig. 1f). The combinative diagnostic capability was better than one of these markers alone. At the optimal cut-off value (1.720), the sensitivity and specificity of exosomal HOTTIP were 69.8 and 85.0%, respectively. When we set the specificity as 95%, exosomal HOTTIP had the highest sensitivity (Additional file 4: Table S1). These results indicated that exosomal HOTTIP might be an appropriate diagnostic marker for GC.

### Prognostic value of exosomal HOTTIP for GC patients

**Fig. 2:**
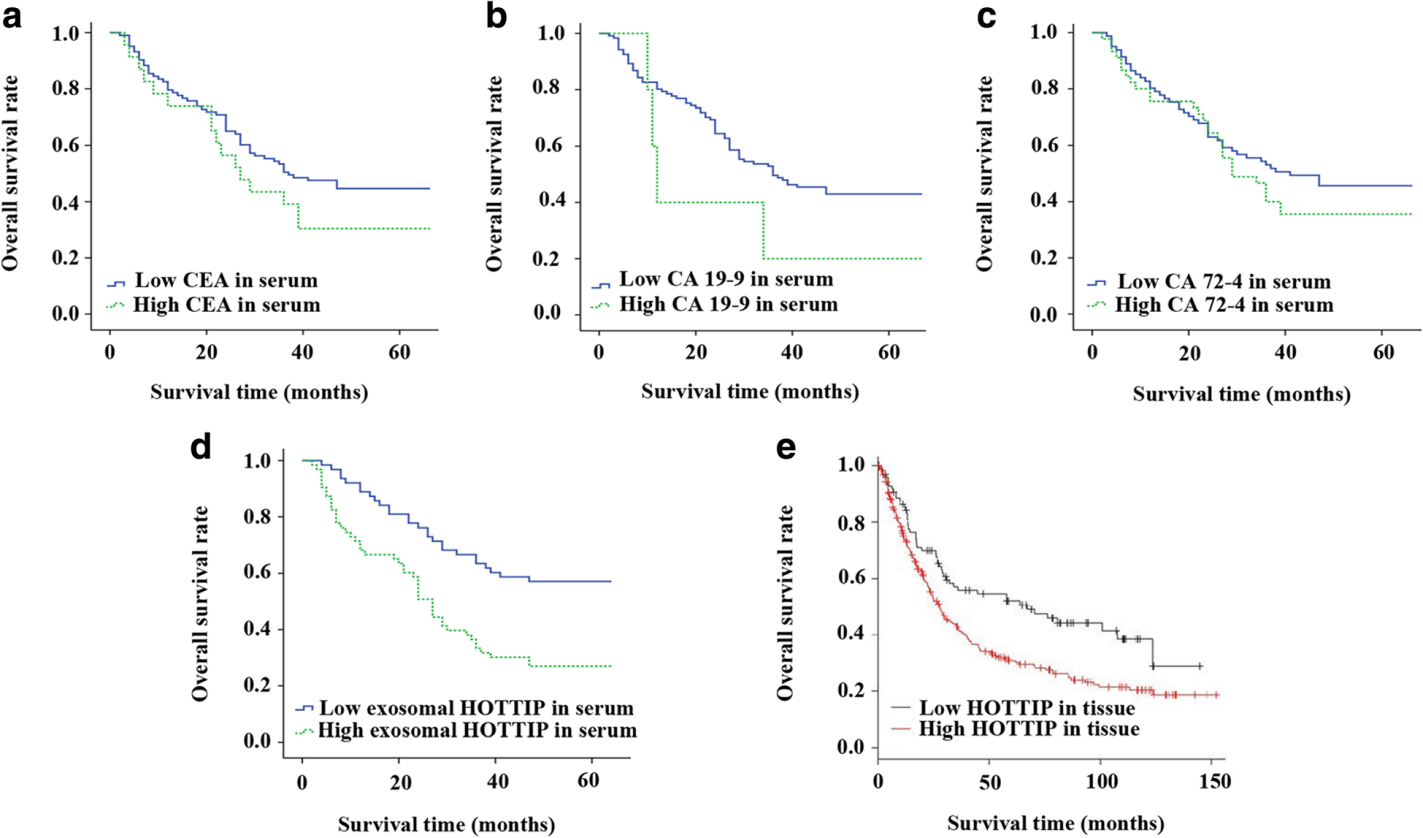
Association between tumor markers and overall survival in gastric cancer. Kaplan–Meier curves of gastric cancer patients according to serum levels of **a** CEA, **b** CA 19–9, **c** CA 72–4 and **d** exosomal HOTTIP, and **e** levels of HOTTIP in tissue

Emerging evidence showed that HOTTIP was upregulated and correlated to poor prognosis in patients with cancers [18]. In our study, Kaplan–Meier method was performed to analyze the relationships between CEA, CA 19–9, CA 72–4 and exosomal HOTTIP expression and overall survival rate of patients with GC. The results demonstrated that there was no significant relationship between these traditional tumor markers and OS (all *P* > 0.05; Fig. 2a, b and c). However, patients with high exosomal HOTTIP expression had a tendency of shorter 5-year overall survival (*P* < 0.001; Fig. 2d).

The online database K-M plotter (www.kmplot.com), basing on the detection data from GC tissues, was used to validate patient survival rate. One thousand and sixty-five tissue samples of GC were included in K-M plotter database and the patients’ informations were assembled from GEO (Affymetrix microarrays only), EGA and TCGA. The mean follow-up time of these patients was 33 months [19]. We computed prognostic value of HOTTIP by using the Affymetrix probe at a meanprobe of 1564069_at, 244553_at and 1564070_s_at and choosing five databases (GSE14210, GSE15459, GSE22377, GSE29272, GSE51105). Finally, 348 patients’ data were employed. The patients were classified into two groups on the basis of various quantile expressions of the proposed biomarker. Meanwhile, the hazard ratio and logrank *P* value were calculated. The result of K-M plotter survival analysis demonstrated that expression levels of HOTTIP were increased above the optimal cutoff point in 71.8% of patients with GC and these patients had a poor overall survival, with the pooled HR of 1.63 (95% CI = 1.19–2.23, *P* = 0.0022) (Fig. 2e), which supported our conclusion.

Cox proportional hazards model were calculated to identify independent prognostic factors. Univariate analysis indicated that advanced TNM stage (HR = 2.000, 95% CI = 1.225–3.264, *P* = 0.006) and high exosomal HOTTIP levels (HR = 2.352, 95% CI = 1.460–3.791, *P* < 0.001) were poor prognostic factors (Additional file 3: Figure S3). Then features with a value of *P* < 0.05 in univariate analysis were put into multivariate analysis. The analysis showed that high exosomal HOTTIP levels were independent factors associated with poor OS of GC (HR = 2.037, 95% CI = 1.085–3.823, *P* = 0.027) (Additional file 5: Table S2).

## Conclusions

Our study reveales the presence of HOTTIP in exosomes isolated from serum samples of GC patients. We find that exosomal HOTTIP is upregulated in the serum of GC patients and it may be a better biomarker for GC in diagnosis than CEA, CA 19–9 and CA 72–4. Furthermore, exosomal HOTTIP expression levels is identified as an independent factor for poor prognosis in GC patients. In conclusion, exosomal long noncoding RNA HOTTIP may be a potential biomarker for gastric cancer in diagnosis and prognosis.

## Additional file

Additional file 1 **Figure S1.** Levels of exosomal HOTTIP expressed in cell culture medium. (PDF 561 kb)

Additional file 2 **Figure S2.** The expression levels of exosomal HOTTIP were upregulated in gastric cancers serum. (PDF 732 kb)

Additional file 3 **Figure S3.** Stability of exosomal HOTTIP levels. Exosomal HOTTIP levels remained stable when treated with a prolonged exposure to room temperature, b and multiple freeze-thaw cycles. (PDF 1778 kb)

Additional file 4 **Table S1.** The sensitivity of biomarkers when the specificity was 95%. (PDF 7 kb)

Additional file 5 **Table S2.** Univariate and multivariate analysis of clinicopathological parameters influencing prognosis. (PDF 90 kb)

## Abbreviations

CA 19–9: Carbohydrate antigen 19–9
CA 72–4: Cancer antigen 72–4
CEA: Carcinoembryonic antigen
GAPDH: Glyceraldehyde-3-phosphate dehydrogenase
GC: Gastric cancer
HOTTIP: The HOXA transcript at the distal tip
UBC: Ubiquitin C
