# Supplementary figures and images for: Exosomal long noncoding RNA HOTTIP as potential novel diagnostic and prognostic biomarker test for gastric cancer
Source: Mol Cancer. 2018 Feb 27;17:68. doi: 10.1186/s12943-018-0817-x (PMC6389063; doi:10.1186/s12943-018-0817-x)

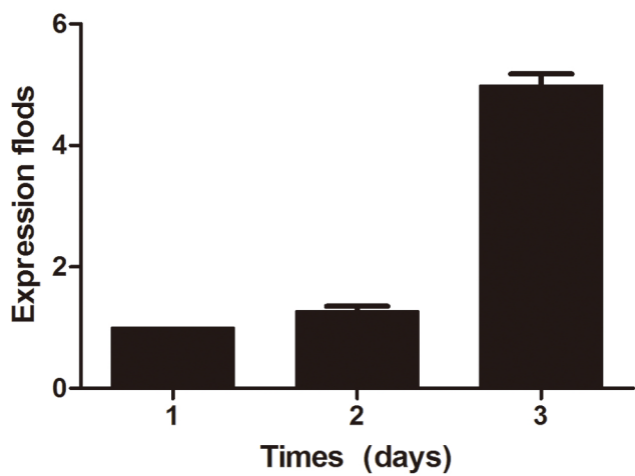

Supplement: Supplementary file 1 — Figure S1. Levels of exosomal HOTTIP expressed in cell culture medium. (PDF 561 kb) [file 12943_2018_817_MOESM1_ESM.pdf]

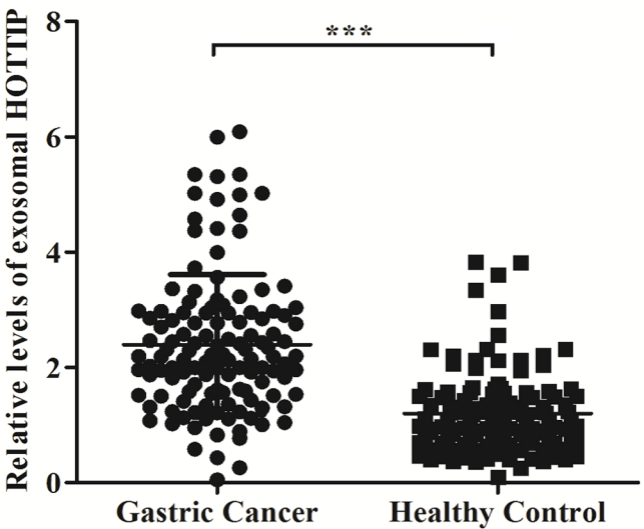

Supplement: Supplementary file 2 — Figure S2. The expression levels of exosomal HOTTIP were upregulated in gastric cancers serum. (PDF 732 kb) [file 12943_2018_817_MOESM2_ESM.pdf]

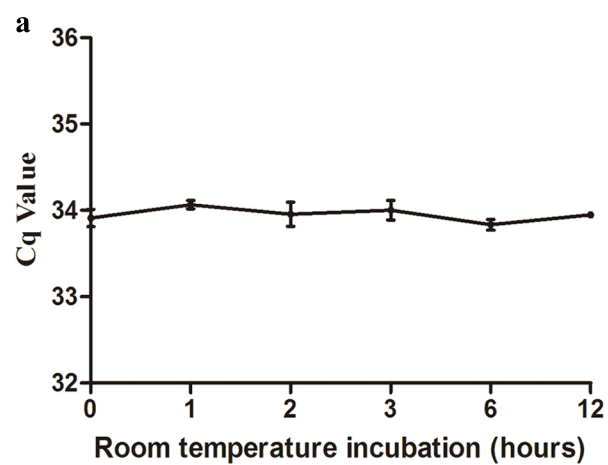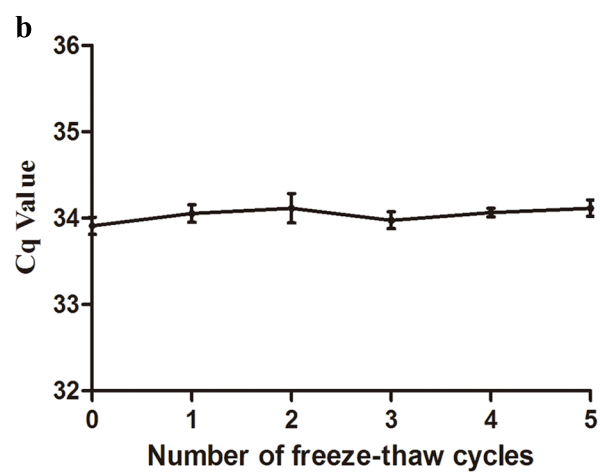

Supplement: Supplementary file 3 — Figure S3. Stability of exosomal HOTTIP levels. Exosomal HOTTIP levels remained stable when treated with a prolonged exposure to room temperature, b and multiple freeze-thaw cycles. (PDF 1778 kb) [file 12943_2018_817_MOESM3_ESM.pdf]
