# Supplementary material for: Exosomal long noncoding RNA HOTTIP as potential novel diagnostic and prognostic biomarker test for gastric cancer
Source: Mol Cancer. 2018 Feb 27;17:68. doi: 10.1186/s12943-018-0817-x (PMC6389063; doi:10.1186/s12943-018-0817-x)
Supplement: Supplementary file 4 — Table S1. The sensitivity of biomarkers when the specificity was 95%. (PDF 7 kb) [file 12943_2018_817_MOESM4_ESM.pdf]

Table S1. The sensitivity of biomarkers when the specificity was 95%

Biological markers	Sensitivity(%)
Exosomal HOTTIP	44.44
CEA	19.84
CA 19-9	10.32
CA 72-4	29.37
CEA+CA 19-9+CA 72-4	39.68
CEA+CA 19-9+CA 72-4+Exosomal HOTTIP	55.56
