# Supplementary material for: Exosomal long noncoding RNA HOTTIP as potential novel diagnostic and prognostic biomarker test for gastric cancer
Source: Mol Cancer. 2018 Feb 27;17:68. doi: 10.1186/s12943-018-0817-x (PMC6389063; doi:10.1186/s12943-018-0817-x)
Supplement: Supplementary file 5 — Table S2. Univariate and multivariate analysis of clinicopathological parameters influencing prognosis. (PDF 90 kb) [file 12943_2018_817_MOESM5_ESM.pdf]

Table S2. Univariate and multivariate analysis of clinicopathological parameters influencing prognosis

Features	univariate analysis		*P* value	multivatiate analysis		*P* value
	HR	95%CI		HR	95%CI	
Age	1.127	0.711-1.785	0.611			
Gender	1.094	0.691-1.733	0.702			
Tumor size	1.072	0.678-1.697	0.765			
Pathological differention	1.173	0.733-1.876	0.507			
Invation depth	1.226	0.764-1.966	0.399			
Lymph nodes metastasis	1.326	0.813-2.161	0.258			
TNM stage	2.000	1.225-3.264	0.006**	1.250	0.655-2.385	0.499
Exosomal HOTTIP	2.352	1.460-3.791	<0.001***	2.037	1.085-3.823	0.027*

HR, hazard ratio; CI, confidence interval. * $P < 0.05$, ** $P < 0.01$, *** $P < 0.001$.
